# Editorial: Biomedical sensing in assistive devices

**DOI:** 10.3389/fbioe.2026.1798992

**Published:** 2026-02-18

**Authors:** Peihua Li, Zhi Pang, Zhi-qiang Zhang, Chong Li, Yanhong Liu, Wujing Cao

**Affiliations:** 1 College of Engineering, Southern University of Science and Technology, Shenzhen, China; 2 Shenzhen Institutes of Advanced Technology, Chinese Academy of Sciences, Shenzhen, China; 3 College of Intelligent Robotics and Advanced Manufacturing, Fudan University, Shanghai, China; 4 University of Leeds, Leeds, United Kingdom; 5 Tsinghua University Beijing, Beijing, China; 6 Hospital of Zhengzhou University, Zhengzhou University, Zhengzhou, China

**Keywords:** assistive devices, biomedical sensing, intention recognition, rehabilitation robotics, wearable robotics

## 1 Introduction

Biomedical sensing in assistive devices represents a dynamic intersection between biomedical engineering and advanced sensor technologies. It aims to enhance the functionality, adaptability, and overall user experience of assistive systems for individuals with disabilities as well as those with general health challenges. These technologies integrate a wide array of sensors, wheelchairs, exoskeletons, and wearable devices. Through these embedded sensing modules, assistive devices are able to capture real-time information about user movements, physiological states, and environmental conditions. This information can then be translated into responsive modifications of device behavior. In turn, such adaptations optimize functional performance and help prevent potential accidents or injuries. Ultimately, biomedical sensing in assistive devices holds significant promise for improving quality of life and overall wellbeing. It paves the way for more personalized, adaptive, and user-centered assistive technologies. Biomedical sensing technologies are fundamentally reshaping the design philosophy and operational paradigm of assistive devices. These systems are evolving from traditional mechanical tools into intelligent platforms capable of perceiving, interpreting, and responding to user needs in real time. The present Research Topic, Biomedical Sensing in Assistive Devices, covers a broad spectrum of innovations—from EMG- and EEG-based physiological signal acquisition to wearable systems, rehabilitation robots, molecular diagnostic tools, and medical image processing methods. Collectively, the twelve contributing articles demonstrate significant advancements in multimodal biosignal analysis, intelligent control strategies, wearable and soft-robotic device evaluation, rapid molecular diagnostics, and deep-learning–based medical image interpretation. Together, they outline the emerging closed-loop paradigm of “sensing–decision making–action execution” in next-generation assistive technologies.

To present these contributions more systematically, this Editorial organizes the articles into four thematic categories: (1) Motion intention recognition and intelligent control; (2) Design and evaluation of wearable and rehabilitation devices; (3) Biomedical diagnostics and fundamental sensing technologies; (4) Medical imaging and signal processing methods.

## 2 Motion intention recognition and intelligent control

Nocilli et al. developed a projection-based myoelectric control algorithm that maps the electromyographic signals of stroke survivors onto physiological reference patterns. The goal is to reduce abnormal co-contraction and promote muscle activation profiles that more closely resemble normal physiology. In preliminary experiments involving three individuals with chronic stroke, the authors observed that the myoelectric control patterns tended to shift toward those of healthy participants. This trend was closely associated with improvements in movement planning and motor accuracy. These findings provide a basis for future longitudinal rehabilitation interventions. Pang et al. proposed an SDA–LSTM deep learning framework that classifies four gait phases in children with cerebral palsy using IMU data. The dataset was collected in real community environments, and the method maintained a high level of accuracy even under substantial noise (97.83%), demonstrating its potential clinical value in CP gait analysis and ankle exoskeleton control. Miao et al. constructed a robot-assisted mirror rehabilitation model based on equivalent kinematics, employing a broaden learning system to sense bilateral movement features and automatically adjust damping. The results indicate that this framework enables individuals with weaker upper-limb strength to perform smoother mirror movements. It also enhances active participation, providing a practical approach for personalized rehabilitation control. Lei et al. developed an intelligent automatic docking system for wheelchairs or hospital beds, integrating LIDAR, IMU, QR-code–based visual positioning, and laser triangulation ranging. The system performs remote navigation, mid-range pose adjustment, and high-precision docking in sequential stages, significantly improving the autonomy and safety of smart nursing equipment. Dong et al. combined a CNN with a transfer learning strategy to extract readiness potential features from EEG motor-related cortical activity, achieving lower-limb movement intention prediction approximately 484 ms in advance. Offline accuracies reached 95% for the right leg and 91% for the left leg, and online accuracy was 82.75%. These results suggest strong applicability for anticipatory intention-based control in exoskeleton systems.

## 3 Design and evaluation of wearable and rehabilitation devices

Almujally et al. integrated the mHealth and ScientISST MOVE datasets and applied machine-learning algorithms to multimodal wearable sensor data. Their approach achieved activity classification accuracies of up to 95%, highlighting the potential of wearable devices for intelligent, home-based health monitoring. Woo et al. proposed a personalized oral–motor rehabilitation device that employs flexible sensors to provide real-time feedback on training intensity while pairing with a mobile application to track tongue movement. Preliminary experiments confirmed the feasibility of this system in managing training intensity and enhancing orofacial muscle performance. This indicates its potential to serve as a more precise and tailored tool for speech rehabilitation. Zhang et al. were the first to use ultrasound imaging to evaluate, in real time, the effect of a soft wearable respiratory assistive robot on diaphragm displacement, and they further established a coupled human–robot respiratory mechanics model. The experiments demonstrated that the assistive device increased diaphragm displacement by 1.95-fold and lung volume by 2.14-fold. These results suggest that the system can enhance ventilation capacity and help prevent disuse-induced diaphragmatic atrophy.

## 4 Biomedical diagnostics and fundamental sensing technologies

Yu et al. developed a one-pot RPA–Cas12a/13a rapid detection assay capable of simultaneously identifying HPV16 and HPV18 within 40 min, with a sensitivity of 10 copies/µL and corresponding sensitivity and specificity of 97.69% and 100%. The method is well suited for primary-level screening and rapid clinical diagnostics. Xu et al. designed a dual-coil electromagnetic sensor to monitor changes in cerebral blood flow and proposed a new conductivity reactivity index, CRx. In animal models, CRx showed a significant negative correlation with the clinically used PRx, indicating that it can noninvasively reflect cerebrovascular reactivity and help estimate the optimal cerebral perfusion pressure. This provides a potential alternative approach for monitoring patients with severe brain injury. Zhang et al. conducted a bibliometric and visualization analysis of 114 publications on tear-based electrochemical biosensors. They reported rapid growth in the field since 2008, with research hotspots centered on “tear glucose,” “disease diagnosis,” and “*in vivo* detection.” The study also identified influential authors and institutions, offering valuable references for future research on ocular and systemic disease biomarkers.

## 5 Medical imaging and signal processing methods

Ding et al. proposed the MARes-Net multi-scale attention residual network, achieving precision and recall rates exceeding 93% in jaw cyst image segmentation. Through the integration of multi-scale feature perception, attention mechanisms, and residual structures, the network demonstrated substantial performance improvements when applied to complex oral imaging. These results highlight its suitability for use in auxiliary diagnosis and preoperative assessment.

The twelve articles in this Research Topic span topics from signal acquisition, sensor design, and robotic control to wearable device evaluation, molecular diagnostics, and medical imaging algorithms, illustrating the trend of assistive technologies toward greater precision, intelligence, and clinical accessibility. Future efforts should focus on several key directions. First, clinical validation of closed-loop assistive systems based on multimodal sensing is essential. Second, achieving low-power, lightweight wearable devices for long-term monitoring remains a priority. Third, intention recognition algorithms must demonstrate robustness across diverse populations and application scenarios. Finally, the integration of non-invasive physiological monitoring and rapid molecular diagnostic technologies with assistive devices warrants further development. Collectively, these studies provide a critical foundation for translating assistive devices from the laboratory into home and clinical settings.

